# A general method for baseline-removal in ultrafast electron powder diffraction data using the dual-tree complex wavelet transform

**DOI:** 10.1063/1.4972518

**Published:** 2016-12-19

**Authors:** Laurent P. René de Cotret, Bradley J. Siwick

**Affiliations:** 1 Department of Physics, McGill University, 3600 rue University, Montréal, Québec H3A 2T8, Canada; 2 Department of Chemistry, McGill University, 801 rue Sherbrooke Ouest, Montréal, Québec H3A 0B8, Canada

## Abstract

The general problem of background subtraction in ultrafast electron powder diffraction (UEPD) is presented with a focus on the diffraction patterns obtained from materials of moderately complex structure which contain many overlapping peaks and effectively no scattering vector regions that can be considered exclusively background. We compare the performance of background subtraction algorithms based on discrete and dual-tree complex (DTCWT) wavelet transforms when applied to simulated UEPD data on the M1–R phase transition in VO_2_ with a time-varying background. We find that the DTCWT approach is capable of extracting intensities that are accurate to better than 2% across the whole range of scattering vector simulated, effectively independent of delay time. A Python package is available.

## I. MOTIVATION

Ultrafast electron diffraction (UED) has matured into a widely applicable technique for investigating photo-induced structural dynamics in gas phase^1,2^ and solid-state samples.^3^ Bulk dynamics in inorganic^4^ and organic^5^ single crystals, polycrystalline,^6,7^ and amorphous materials^8^ have been studied. Surface structural dynamics have also been interrogated using reflection geometries.^9,10^ Many of these remarkable successes have, directly or indirectly, been built on the efforts of Zewail and his group whose pioneering contributions are being celebrated through this special issue. Zewail was one of the very first to fully appreciate the profoundly new window on atomic-level structural dynamics that ultrafast electron probes could provide.

Preparing electron transparent single crystal samples of sufficient quality, quantity, and size for UED experiments is one of the bottlenecks that prevent higher experimental throughput. Ultramicrotomy^11^ and exfoliation have proven effective for layered materials, but non-layered materials present significant difficulties. By comparison, pulsed laser deposition (PLD), sputtering and other thin-film deposition techniques can readily produce very high quality polycrystalline samples for a wide variety of material systems^12^ on electron-transparent substrates with controlled thickness.^13^ Such polycrystalline samples have the potential to considerably broaden the range of materials systems accessible to investigation with UED by bypassing difficulties associated with producing electron transparent single crystals of non-layered materials.

Polycrystalline samples probed by UED present different data processing challenges than single crystal samples due to the projection of the three-dimensional reciprocal space into a single (radial) dimension; most importantly, Bragg diffraction peaks that are well-separated in the Brillouin zone can overlap in polycrystalline data. Moreover, potentially time-dependent background signals—from inelastic scattering to diffraction from the substrate—can interfere with dynamics in the Bragg peaks.

Baseline-removal in UED has previously been handled either by curve-fitting biexponentials,^7^ bilorentzians,^14^ or by polynomial interpolation.^15^ These techniques are reliable only in the case of highly-symmetric crystal structures and small unit cells, which yields polycrystalline diffraction patterns with well-defined background-only scattering angle regions. Fourier analysis methods fail because there is significant overlap in the frequency domain between the background and elastic scattering signals.

Wavelet transforms are more suitable as an analysis tool of finite-sized data because their basis functions are localized in space and frequency, unlike those of the Fourier transform. Baseline-removal techniques based on the discrete wavelet transform (DWT) are regularly used in other fields, for example, in removing background in surface-enhanced Raman spectroscopy^16^ and polycrystalline x-ray diffraction.^17^ However, while DWT-based approaches are more robust than polynomial interpolation for baseline-determination, it also presents some drawbacks; in particular, DWT wavelet coefficients tend to oscillate around sharp signal features, and aliasing artefacts can appear due to the use of real-valued wavelets.^18^

The dual-tree complex wavelet transform (DTCWT) improves on the DWT by eliminating both of these drawbacks, and introduces near-shift-invariance.^18^ The DTCWT has been shown to outperform the DWT in baseline-removal of energy-dispersive x-ray fluorescence spectra.^19^

## II. METHODS

The DWT, implemented using Mallat's algorithm, consists of the application of two related filter banks to the data, a low-pass filter and a high-pass filter, to produce so-called approximate (low-pass) coefficients and detail (high-pass) coefficients, as is well-described by Mallat in Ref. 20. Multiple levels of the transform are obtained by iteratively transforming the approximate coefficients from the previous level. The approximate coefficients are of interest to baseline-determination because it contains the local low-frequency components of the signal.

The DTCWT is an extension of the DWT from real-valued wavelets to complex-valued wavelets, which in the discrete case are represented with four related filter banks (rather than two). Each tree is an application of the DWT, one for each part (real and imaginary) of the wavelet. The approximate and detail coefficients are then combined into complex form. Interested readers will find more information Ref. 21.

**FIG. 1.**
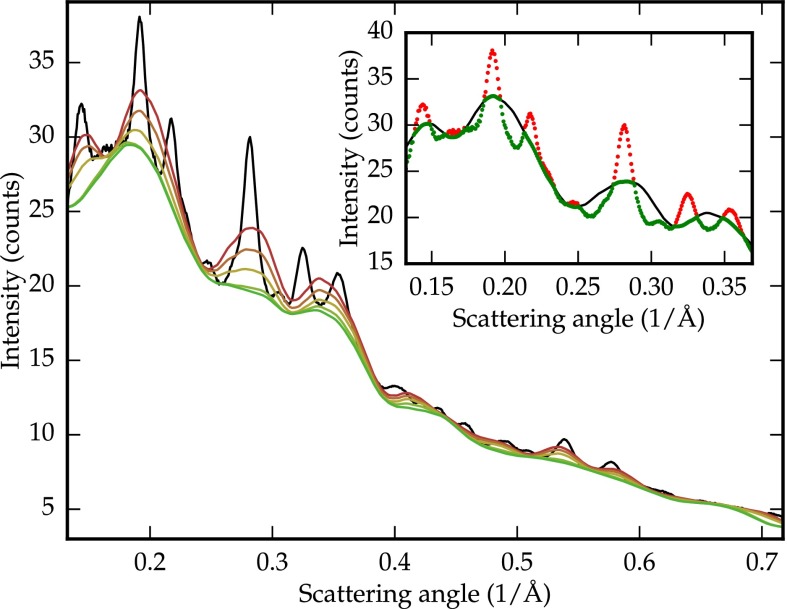
Baseline-determination on real data after 1, 2, 5, 50, and 150 iterations. Inset: at each iteration, the signal (red) above the baseline (black) is rejected as being part of a peak. The next iteration is run on the remaining signal (green).

Bragg diffraction peaks present in UED data have a Voigt profile, with a theoretically infinitely wide frequency band. Therefore, truncating the wavelet coefficients obtained from UED data to isolate the background will inevitably lead to loss of information in the diffraction peaks as well. This frequency overlap between the background and peaks mandates the use of an iterative approach that progressively remove the influence of peaks, until only the baseline is left. Such an iterative algorithm, based on the DWT, was presented by Galloway *et al.* in Ref. 16. We present the extension of this algorithm to the use of the DTCWT (Fig. 1) and compare the performance of the algorithm using both transforms on simulated ultrafast electron powder diffraction (UEPD) datasets with known dynamical baselines.

## III. RESULTS

The test of validity comes from imposing a known, time-varying baseline to a simulated ultrafast electron diffraction experiment. The simulated data represents the insulator-to-metal transition in vanadium dioxide. The transition between the insulating monoclinic phase (M1) and its metallic rutile phase (R) is achieved by computing two simulated diffraction patterns, *I*_M1_ (*s*) and *I*_R_ (*s*), and combining them with a time constant *τ* = 200 fs

$$I(s)\propto e^{-t/\tau }I_{M1}(s)+(1-e^{-t/\tau })I_{R}(s).$$ (1)

**FIG. 2.**
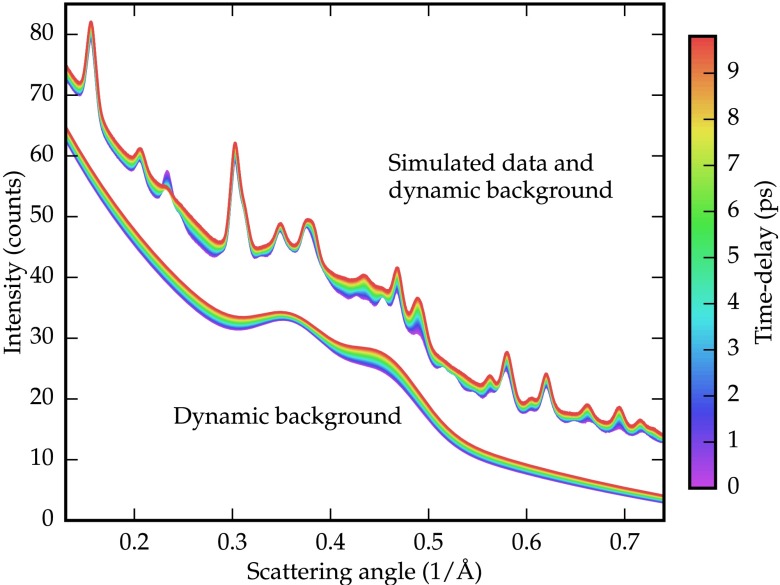
Simulated dataset representing the ultrafast structural phase transition of vanadium dioxide from rutile to monoclinic, with a time-varying background. Two Gaussian bumps are representative of diffraction experiments on silicon nitride substrates. The isolated background is shown offset by −10 counts.

The simulated patterns are convolved with the instrumental broadening parameters determined from the data presented in Ref. 7. A biexponential background is added, supplemented with two Gaussian bumps typical of experiments involving silicon nitride substrates. The parameters determining the biexponential base and the Gaussian bumps are varied over time by up to 10%, linearly, in opposite directions. Finally, Gaussian noise with a standard deviation of 5% of the maximal peak intensity at time zero is added to all signals. The resulting dataset is presented in Figure 2.

**FIG. 3.**
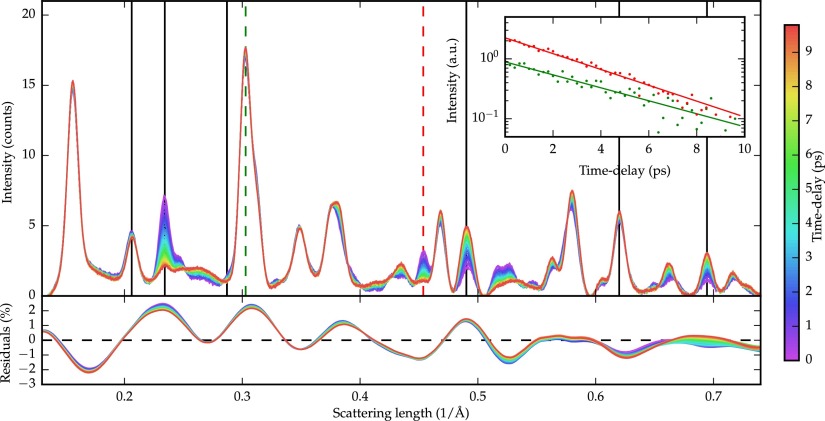
Top: Unassisted reconstruction of noisy signal from simulated time-varying raw data. The powder diffraction patterns are characteristic of the photoinduced insulator-to-metal transition of VO_2_. Bottom: Error on the baseline-determination over time. Baseline-determination error evolution over time is bounded at 1%, which is crucial for correct peak dynamics measurements. Inset: Exponential fit extracted from peaks highlighted by colored (dashed) vertical lines.

The baseline is determined individually for each time-delay using the algorithm described in Section II. The baseline-subtracted signals are shown in Figure 3, along with the relative error in the baseline-determination over time. While the absolute error in the baseline-determination can go up to 2%, we note that the dynamics in the error are capped at 1% change over time. UED experiments are more concerned with the change in signal over time, and therefore, a near-constant relative error would not obscure dynamics by decreasing the effective signal-to-noise ratio in the peak intensity dynamics.

Part of the power of ultrafast experiments resides in its effectiveness at isolating different processes by identifying time-constants; a good baseline-removal technique must leave the dynamics intact in this regard. With this in mind, the time constant of the transition is extracted from the baseline-subtracted signals. The peaks indicated by lines in Figure 3 are fit over time with an exponential function, and the average time constant was found to be *τ* = (200 ± 15) fs, in perfect agreement with the simulation time constant *τ* = 200 fs, despite the fact that the background time-dependence is not exponential. Moreover, the average error in amplitude change over fitted peaks was found to be 1%. Peak dynamics highlighted by colored (dashed) line are presented in the inset of Figure 3, along with exponential fits showcasing the quality of recovered dynamics. Despite the non-exponential character of the background dynamics, the peaks show no visible distortion.

**FIG. 4.**
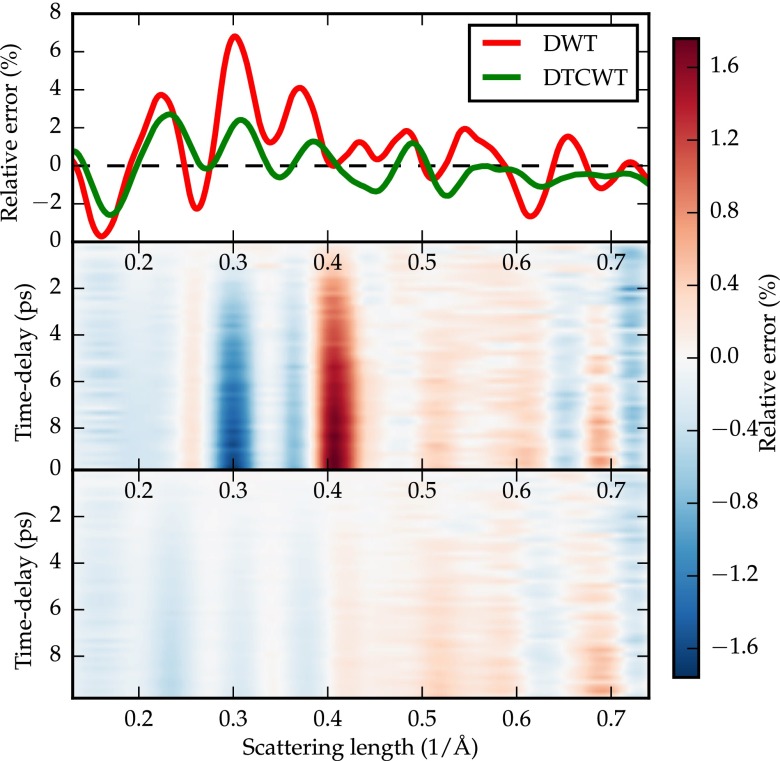
Top: Baseline-determination relative error at time *t* = 0 using the two techniques described. Middle: Evolution of the relative error of the DWT-based algorithm as a function of time, referenced to *t* = 0. Bottom: Evolution of the relative error of the DTCWT-based algorithm as a function of time, referenced to *t* = 0.

It is informative to compare the results given by the DTCWT with the DWT. We use the same algorithm, with the only difference being the use of the DTCWT or DWT at each step. Figure 4 shows the residuals at time-delay *t* = 0 of the baseline-determination using both the DWT and DTCWT, as well as the evolution of the residuals over time. The comparison to the DWT is advantageous to the DTCWT in two aspects: the error signal at time-delay *t* = 0 is smaller in amplitude and the evolution of the error is minimal—crucial for a high signal-to-noise ratio. The RMS error across scattering length varies between 1.06% and 1.15% over time for the DTCWT, and between 2.04% and 2.30% over time for the DWT. This two-fold improvement demonstrates that a baseline-removal algorithm based on the DTCWT should be preferred to the use of the DWT in the case of UED experiments.

## IV. CONCLUSION

A baseline-removal algorithm for ultrafast electron diffraction, based on the dual-tree complex wavelet transform, was shown to outperform other techniques in the case of non-trivial crystal structures, where background-only regions cannot be identified. The intrinsic dynamics of samples were extracted without distortion, despite greatly exaggerated noise levels and background dynamics. The algorithm requires minimal user input, and therefore integrates well into automatic processing routines.

The algorithm presented herein can be extended to the baseline-removal of (2D) single crystal UED data, paving the way for reliable analysis of complex organic structures for which diffraction peaks overlap significantly.

An open-source Python package implementing the transforms and algorithms described in this work is available on the authors' website.^22^
